# Impact of nursing home admission on health care use and disease status elderly dependent people one year before and one year after skilled nursing home admission based on 2012–2013 SNIIRAM data

**DOI:** 10.1186/s12913-017-2620-6

**Published:** 2017-09-18

**Authors:** A. Atramont, I. Bourdel-Marchasson, D. Bonnet-Zamponi, I. Tangre, A. Fagot-Campagna, P. Tuppin

**Affiliations:** 1 Caisse Nationale d’Assurance Maladie fund (CNAM-TS), Direction de la Stratégie des Études et des Statistiques, 26-50, avenue du Professeur André Lemierre, F-75986 Paris Cedex 20, France; 2 Clinical gerontology department, Centre Henri Choussat, Xavier Arnozan Hospital, 33604 Pessac cedex, France; 3 Centre for pharmacoepidemiology (APHP), Paris, France; 4 OMEDIT: Observatoire des MEdicaments Dispositifs médicaux et Innovations Thérapeutiques d’Ile de France, Paris, France; 5 Korian Bonisiaca nursing home, 93140 Bondy, France

**Keywords:** Nursing home, Disease status, Healthcare use, Health insurance database, Sniiram

## Abstract

**Background:**

The aim of this study was to compare disease status and health care use 1 year before and 1 year after skilled nursing home (SNH) admission.

**Methods:**

People over the age of 65 years admitted to SNH during the first quarter of 2013, covered by the national health insurance general scheme (69% of the population of this age), and still alive 1 year after admission were identified (*n* = 14,487, mean age: 86 years, women: 76%). Their reimbursed health care was extracted from the *Système National d’Information Interrégimes de l’Assurance Maladie* (SNIIRAM) [National Health Insurance Information System].

**Results:**

One year after nursing home admission, the most prevalent diseases were cardiovascular/neurovascular diseases and neurodegenerative diseases (affecting 45% and 40% of people before admission vs 51% and 53% after admission, respectively). Physical therapy use increased (43% vs 64% of people had at least one physical therapy session during the year, with an average of 47 vs 84 sessions/person during the year), while specialist consultations decreased (29% of people consulted an ophthalmologist at least once during the year before admission vs 25% after admission; 27% vs 21% consulted a cardiologist). Hospitalization rates were lower during the year following institutionalization (75% vs 40% of people were hospitalized at least once during the year), together with a lower emergency admission rate and a higher day admission rate.

**Conclusions:**

*A*nalysis of the new French reimbursement database specific to SNH shows that nursing home admission is associated with a reduction of some forms of outpatient care and hospitalizations.

**Electronic supplementary material:**

The online version of this article (10.1186/s12913-017-2620-6) contains supplementary material, which is available to authorized users.

## Background

France, like many countries, is faced with the problem of aging of the population. On 1st January 2013, 11.5 million people 65 years and older represented 17.6% of the 65.6 million inhabitants of France and people 75 years and older represented 9% of this population [1]. If current trends continue, almost one in three people will be over the age of 60 years in 2060 [2]. In parallel, the number of dependent elderly people is increasing: 1.2 million people (7.8% of people 60 years and older) received the *Allocation Personnalisée d’Autonomie* (APA) (dependent person’s pension) in metropolitan France in 2012, with a predicted 2.3 million in 2060 [3].

In this context, a cross-sectional survey conducted every 4 years (2007, 2011, 2015) by the French Ministry of Health among *Établissements d’Hébergement pour Personnes Âgées skilled* nursing homes (EHPA survey) provides the main data on the numbers and clinical characteristics of institutionalized elderly people [4–6]. Almost 700,000 elderly people were living in a nursing home at the end of 2011, i.e. 10% of the population 75 years and older and almost 23% of the population 85 years and older, and 573,600 (83%) of these people lived in specific *Établissements d’Hébergement pour Personnes Âgées Dépendantes* (EHPAD) [Skilled nursing homes (SNH)] [6]. The 2011 EHPA survey on all SNH residents, reported 26% prevalence for arrhythmias and conduction disorders, 18% for coronary heart disease, 18% for stroke and 49% for dementia [7]. Thus, detailed information on health care use and disease status of institutionalized elderly people are usually derived from specific cross-sectional surveys conducted among physicians, administrative personnel and residents of a sample of institutions, based on relatively small sample sizes with or without comparisons with elderly people living at home. Analysis of trends before and after nursing home admission would require specific prospective studies, which would be more difficult and expensive to perform.

In France, SNH transmit the lists of their residents to national health insurance, which can be linked to data of the National Health Insurance Information System, which comprises information concerning reimbursed or free health care use. The objective of this observational study was to compare, by linking these two databases, disease status and health care use of national health insurance general scheme beneficiaries between the year before nursing home admission and the year after nursing home admission.

## Methods

### Data sources

In France, there were about 7500 SNH with 600,000 residents in 2011. SNH are available to dependent elderly people 60 years and older and admission is based on the choice of the person and not mainly on the dependency level. Nevertheless, there are very few persons between 60 and 64 years-old in SNH. The RESID-EHPAD tool allows these SNH to automatically transmit the lists of their residents with admission and discharge dates to French national health insurance. Information concerning these institutions is also collected, which determines the types of health care covered by the lump sum paid to the nursing home by French national health insurance: partial or global budget payment system, presence or absence of a nursing home pharmacy. RESID-EHPAD data are available with a history of 33 sliding months and can be linked to the SNIIRAM database by means of a common identifier.

The *Système National d’Information Interrégimes de l’Assurance Maladie* (SNIIRAM) [National Health Insurance Information System] database anonymously and comprehensively collects individual data for all health care reimbursed over a three-year period plus the current year to the beneficiaries of the various health insurance schemes [8]. It does not contain any information concerning clinical results related to prescriptions or examinations. However, it contains information concerning costly long-term diseases (ALD) status, validated by a national health insurance physician at the request of the attending physician, allowing exemption of copayment, can provide information about the nature of the diseases treated. An anonymous and unique identification number for each beneficiary allows this information to be linked to the data collected during hospital stays in the various types of health care institutions by the *Programme de médicalisation des systèmes d’information* (PMSI) [Hospital discharge System]. Hospital diagnoses are coded according to the International Classification of Diseases 10th Edition (ICD-10), in the same way as the diagnoses allowing attribution of ALD status.

Nevertheless, all health care delivered to SNH residents can be identified in the SNIIRAM database depending on the nursing home’s mode of funding, as nursing care is included in the global budget paid to all SNH. For SNH opting for the partial budget payment system, general practitioner and physical therapist fees are reimbursed individually and can therefore be identified. Drug reimbursements can also be identified individually in the absence of a nursing home pharmacy.

### Population

**Fig. 1:**
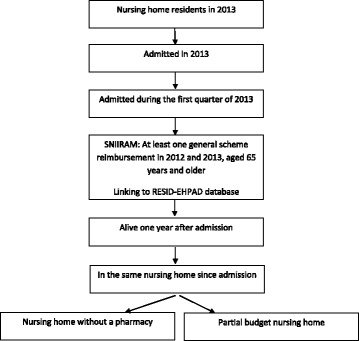
Selection of the study population from RESID-EHPAD and SNIIRAM databases

This study focused on people aged 65 years and over, covered by the French national health insurance general scheme, the main health insurance scheme in France (covering about 69% of the French population of this age), admitted to SNH during the first quarter of 2013, and presenting at least one reimbursed health care use in both 2012 and 2013 (Fig. 1). Comparison between the year before and the year after SNH admission required the study population to be limited to residents who were still alive 1 year after SNH admission and who had not changed SNH (in order to maintain the same type of payment system). This was the case for specialist physician and dental consultations and short-stay hospitalizations (*n* = 14,887). Nevertheless, for specific analysis, the study population was therefore limited to certain types of SNH according to the types of individually reimbursed health care. Assessment of the residents’ disease status in 2012 and 2013 was based on residents admitted to a nursing home without a pharmacy, as some disease identification algorithms take drug reimbursements into account (*n* = 11,687). Analysis of general practitioner consultations and physical therapy sessions was based exclusively on residents of SNH that had opted for the partial budget payment system (*n* = 10,404).

### Statistical analysis

Data concerning the residents’ diseases were derived from the beneficiaries’ disease and expenditure mapping established for 2012 (before nursing home admission) and 2013 (year of nursing home admission) by CNAM-TS based on SNIIRAM data (Additional fi﻿le 1). This tool can be used to identify diseases and health states in the population of beneficiaries requiring specific management and consequently able to be detected in the SNIIRAM database [9, 10]. Algorithms are used to identify 56 non-exclusive groups of diseases, grouped into 13 main categories, based on principal diagnoses, related or significant associated diagnoses in short-stay hospitals and psychiatric hospitals; ALD status; dispensing of specific drugs; and specific procedures. Schematically, for certain cardiovascular and neurovascular diseases, the term “acute” refers to hospitalization during the year with an appropriate ICD-10 code and the chronic phase is defined by attribution of ALD status during the year and/or hospitalization with specific codes during the last 5 years. The acute episode always takes precedence to a chronic phase and these two groups are mutually exclusive for a given disease. Active cancer is defined by short-stay hospitalizations over a 2-year period (specific diagnoses of cancer and chemotherapy and radiotherapy codes) and/or ALD status attributed over the previous 2 years. Cancer under surveillance is defined from specific hospital codes over a 5-year period and/or ALD status. A cancer is therefore considered to be active when it is responsible, over a 2-year period (year n or n-1), for hospitalization for treatment with the exclusion of hospitalizations for assessment only, or hospitalization for metastasis, or attribution of ALD status, or treatment by certain targeted therapies (dispensed at least 3 times during year n or n-1). A cancer classified as active takes precedence to a cancer of the same site under surveillance.

Consultations with physicians, dentists, physical therapists and hospitalizations were defined by the presence of at least one specific reimbursement during the year before or after nursing home admission. In France, all national health insurance beneficiaries are required to specify a general practitioner to coordinate health care with lower reimbursement rates when a general practitioner is not specified. The change of general practitioner was analyzed by comparing the last general practitioner consulted before nursing home admission and the last general practitioner consulted during the 12 months following nursing home admission. Specialist consultations included outpatient visits in short-stay hospitals as well as office visits.

Admissions to short-stay hospitals were studied by excluding stays corresponding to treatment sessions (dialysis, radiotherapy, chemotherapy, etc.). The reason for admission was defined by the principal diagnoses of the stay and only reasons (section and 3-digit code of ICD-10), representing a total of at least 2% of all hospitalizations before or after nursing home admission. The proportions of day-only or emergency hospitalizations for each of these reasons were reported. Emergency hospitalization was defined by hospitalizations from home (or the nursing home) via the emergency room or direct intensive care unit admission.

Chi-square test, McNemar’s test and t-test were used to compare indices before and after admission and between groups.

Use of SNIIRAM data by the CNAM-TS has been approved by decree and the CNIL (French data protection authority). Analyses were performed using SAS 9.3 software (associated with SAS Enterprise Guide version 4.3).

## Results

### Populations and characteristics

For the 14,887 people included, mean age was 86 years and 76% were women (women 85 years and older represented 51% of the study population). About 70% of nursing home residents (*n* = 10,404) lived in a nursing home opting for the partial budget payment system and 79% (*n* = 11,687) lived in a nursing home without a pharmacy. Their characteristics in terms of age, sex and identified diseases did not differ significantly according to these various subgroups.

### Diseases

In 2012, before admission to a nursing home without a pharmacy (*n* = 11,687), 45% of the population presented a marker for cardiovascular or neurovascular disease: 21% for arrhythmia or cardiac conduction disorder, 14% for coronary heart disease, 12% for heart failure and 12% for stroke (Table 1). Diabetes was identified in 16%, dementia in 35%, psychiatric disorders in 14%, chronic respiratory disease in 11% and cancer in 16% (active cancer: 5%). The prevalence of cardiovascular and neurovascular diseases, except for stroke, increased with increasing age of the residents for both sexes. Dementia was more prevalent in people aged 75–84 years for both sexes.

**Table 1:** Diseases identified in people admitted to skilled nursing homes by sex and age

	Women						Men						Total	
Age (years)	65–74		75–84		≥ 85		65–74		75–84		≥ 85			
*N*	404		2450		6015		340		936		1542		11,687	
	2012	2013	2012	2013	2012	2013	2012	2013	2012	2013	2012	2013	2012	2013
Parameters	%	%	%	%	%	%	%	%	%	%	%	%	%	%
Cardiovascular and neurovascular disease	32.2	32.9	35.7	41.4***	44.0	52.1***	45.0	47.6	56.6	59.9	56.7	65.0***	44.6	51.4***
Acute	11.4	6.4*	7.1	7.1	7.9	10.6***	14.7	5.6***	9.6	6.9*	8.9	9.8	8.4	9.2*
Chronic	27.5	30.4	33.5	39.0***	42.1	49.9***	38.5	45.9	53.7	58.7*	54.7	63.3***	42.3	49.3***
Coronary heart disease	9.4	8.7	10.0	11.5	12.6	14.5**	15.6	16.2	22.9	22.9	22.0	23.6	14.1	15.6**
Acute coronary syndrome	NA	NA	0.6	0.6	0.6	0.6	NA	NA	NA	NA	0.9	0.6	0.6	0.6
Chronic coronary heart disease	8.9	8.2	9.4	10.9	12.0	13.9**	14.4	15.6	22.5	22.4	21.1	23.0	13.5	15.0***
Stroke	17.3	19.1	12.5	14.8*	10.1	13.1***	23.5	22.6	17.8	19.0	13.4	16.6*	12.3	14.8***
Acute stroke	8.2	4.0*	4.0	2.8*	3.2	3.1	10.9	NA	5.4	2.2***	3.5	2.5	4.0	2.9***
Stroke sequelae	9.2	15.1*	8.5	12.0***	6.8	10.0***	12.6	20.3**	12.4	16.8**	9.9	14.1	8.3	12.0***
Heart failure	6.2	6.2	8.7	10.3	13.3	18.9***	10.0	12.4	12.8	16.0*	14.7	20.9***	12.1	16.5**
Acute	3.2	NA	2.4	3.1	3.9	6.6***	3.8	NA	3.2	4.2	4.3	6.2***	3.5	5.3***
Chronic	3.0	4.7	6.3	7.2	9.4	12.3***	6.2	10.0	9.6	11.9	10.4	14.8*	8.6	11.2***
Peripheral arterial occlusive disease	3.7	3.7	4.7	4.9	5.1	5.8	7.9	7.9	10.3	10.0	9.0	9.9***	6.0	6.5
Arrhythmias or conduction disorders	10.4	9.9	15.8	18.9**	21.6	25.9***	11.8	11.2	23.6	25.7	27.7	33.7***	20.7	24.5***
Valvular heart disease	3.7	NA	3.8	3.6	5.4	5.7	NA	NA	4.1	4.5	4.7	5.5	4.7	4.9
Diabetes	17.8	20.0	18.4	18.9	13.3	13.9	20.6	23.2	23.9	24.9	17.4	17.8	16.2	16.8
Insulin-treated	5.9	8.4	6.0	6.7	4.2	5.1*	5.9	10.0*	7.3	9.0	5.1	6.3	5.0	6.2***
Non-insulin-treated	11.9	11.6	12.5	12.3	9.2	8.8	14.7	13.2	16.7	15.9	12.3	11.5	11.1	10.7
Cancer	10.6	11.9	13.6	14.0	13.0	13.7	12.6	14.1	23.0	25.4	25.2	26.7	15.4	16.4*
Active	4.5	4.7	4.0	4.3	4.0	4.4	5.0	5.0	8.5	10.8	11.3	12.3	5.4	6.0*
Under surveillance	6.7	7.7	10.1	10.1	9.2	9.6	8.5	9.1	15.3	16.1	15.0	15.6	10.5	10.9
Breast	3.7	4.2	6.4	6.4	4.5	4.5	–	–	–	–	–	–	3.8	3.8
Colon	NA	NA	1.6	1.6	2.2	2.5	NA	NA	3.4	3.7	3.1	3.2	2.3	2.5
Prostate	–	–	–	–	–	–	3.8	3.2	10.1	11.3	11.2	11.6	2.4	2.5
Psychiatric disorders	39.6	43.1	21.7	23.7	9.0	11.6***	37.1	41.5	18.3	22.8*	7.5	10.6**	14.1	16.8***
Psychotic disorders	13.1	14.1	3.8	4.1	1.1	1.3	13.2	12.9	2.2	2.7	NA	0.7	2.4	2.7
Neurotic and mood disorders	21.5	24.3	13.3	14.4	5.2	6.9***	10.9	13.8	8.8	10.1	3.9	5.0	7.7	9.3***
Bipolar and manic disorders	5.7	6.7	2.4	2.7	0.6	0.6	NA	3.8	1.4	1.5	NA	NA	1.2	1.4
Depression, other mood disorders	13.1	14.4	8.2	9.0	3.1	4.4***	5.9	7.1	4.8	5.9	2.5	3.4	4.6	5.8***
Neurotic disorders	6.2	7.9	4.8	4.9	2.0	2.5	3.8	3.5	3.8	4.1	1.4	1.6	2.8	3.2
Substance abuse disorders	5.9	5.0	1.1	1.1	0.1	0.1	8.2	8.8	1.6	1.6	NA	NA	0.9	0.9
Neurological or degenerative diseases	46.5	52.7	51.9	64.2***	35.4	48.1***	37.6	45.0	52.2	62.0***	33.6	48.9***	40.4	52.8***
Degenerative diseases	38.9	44.8	49.9	62.2***	34.6	47.0***	26.5	33.8*	50.3	60.0***	32.2	47.3***	38.6	50.8***
Dementia	34.9	41.1	44.3	56.6***	32.4	44.5**	22.1	28.5	42.3	52.0***	27.6	42.9***	34.8	46.9***
Parkinson’s disease	7.9	8.9	8.2	9.5	3.4	4.1	9.1	10.9	14.9	17.2	6.6	7.7	6.1	7.1**
Neurological diseases	10.6	12.4	3.3	4.3	1.7	2.5**	13.8	15.3	4.4	4.8	2.3	3.0	3.0	3.8**
Epilepsy	4.7	6.4	1.6	2.5*	0.9	1.6***	8.8	9.7	2.8	3.2	1.5	2.0	1.7	2.4***
Chronic respiratory diseases	10.1	9.2	10.1	10.5	10.3	12.3	16.2	16.2	14.5	16.0	13.8	16.7*	11.2	12.8***
Inflammatory or rare diseases or HIV or AIDS	4.0	3.5	4.7	4.7	3.2	3.5	NA	2.9	3.0	2.7	2.3	3.0	3.4	3.6
Chronic inflammatory diseases	3.2	2.7	4.2	4.3	2.9	3.1	NA	NA	2.4	2.1	1.9	2.4	2.9	3.1
Rheumatoid arthritis	NA	NA	2.2	2.2	1.7	1.7	NA	NA	NA	NA	1.0	1.1	1.5	1.6
Diseases of the liver or of pancreas	3.7	2.5	1.9	2.0	1.4	1.4	4.4	3.5	2.7	3.5	1.4	1.6	1.8	1.8
Other chronic diseases	11.4	13.6	10.1	11.6	12.4	14.8***	9.1	11.5	7.5	8.2	7.3	9.4*	10.7	12.8***

*NA* data not available for sample sizes <10

Comparison before and after admission ***p < 0.001, ** < 0.01,* *p* < 0.05

For parameters definition, see Additionnal file 1

A significant increase after admission was observed for many cardiovascular or neurovascular disease (45% to 51%), dementia (35% to 47%), and also for insulin-treated diabetics (5% to 6%), depression and other mood disorders (5% to 6%), Parkinson’s disease (6% to 7%) and chronic respiratory disease (11% to 13%). A significant increase was found after admission for many cardiovascular or neurovascular diseases for women aged 85 years and older and also for depression. Prevalence increase for dementia was also significant for people aged 75 years and older and for both sexes.

### Outpatient care

Among the 10,404 (70%) residents admitted to SNH opting for a partial budget payment system, at least one general practitioner consultation during the year was observed for 95% of residents before and after nursing home admission (Table 2). The mean number of consultations per year increased from 11 to 15 during the year following admission. A smaller proportion of the youngest residents (65–74 years) consulted a general practitioner (81% consulted a general practitioner both before and after nursing home admission vs 91 and 93% of residents aged 75 to 84 years and 85 years and older, respectively). Almost one-half (46%) of the residents for whom at least one general practitioner consultation was identified 1 year before and 1 year after nursing home admission changed general practitioner during the year following nursing home admission.

**Table 2:** Health care use the year before and the year after admission by age

Age (years)	65–74			75–84			> 85			Total		
Partial budget nursing home N	654			3002			6748			10,404		
All types *N*	1007			4373			9507			14,887		
	Before	After	Before and after	Before	After	Before and after	Before	After	Before and after	Before	After	Before and after
	%	%	%	%	%	%	%	%	%	%	%	%
Partial budget												
General practitioner	86.7	91.5	80.9**	94.4	95.4	91.5*	96.0	95.7	93.0	94.9	95.4	91.8
Physical therapist	31.1	54.6	25.4***	42.8	61.5	35.1***	44.1	66.7	36.4***	42.9	64.4	35.3***
All types												
Ophthalmologist	21.4	21.8	8.0***	29.9	26.0	13.3***	29.3	24.6	13.2***	28.9	24.9	12.9***
Cardiologist	18.2	17.9	6.8***	27.3	20.7	10.6***	27.8	21.8	11.5***	27.0	21.2	10.9***
Neurologist	16.6	10.3	5.3***	17.8	10.2	5.6***	8.3	4.9	1.7***	11.7	6.8	3.1***
Dermatologist	5.4	4.9	NA	7.7	7.0	1.8	8.3	8.7	2.1	7.9	7.9	1.9
Rheumatologist	2.3	2.0	NA	6.1	2.4	1.1***	5.4	2.1	0.9***	5.4	2.2	0.9***
Gastroenterologist	7.3	5.6	1.9	6.4	5.0	1.4**	4.5	3.3	0.5***	5.3	4.0	0.9***
Psychiatrist/Neuropsychiatrist	12.7	10.3	5.9*	8.2	6.1	3.0***	2.6	2.9	0.7	4.9	4.3	1.7**
Pulmonologist	4.4	5.4	2.3	4.3	3.8	1.7	3.5	3.2	1.0	3.8	3.5	1.3
Endocrinologist	2.7	1.7	NA	2.6	1.6	0.6***	1.7	1.1	0.4***	2.0	1.3	0.5***
Nephrologist	2.6	2.8	1.2	1.9	1.8	0.9	1.2	1.2	0.6	1.5	1.4	0.7
Dentist	18.1	21.0	7.0	23.0	22.7	9.5	20.7	20.1	8.3	21.2	21.0	8.6

*NA* data not available for sample sizes <10

Comparison before and after admission ***p < 0.001, ** < 0.01,* *p* < 0.05

A smaller number of residents consulted a specialist during the year following admission compared to the year before admission, but with differences according to specialties (Table 2). A significant decrease was observed for ophthalmologists (29% of residents during the year before admission, 25% during the year after admission), cardiologists (27% before admission, 21% after admission) and neurologist (12% before admission, 7% after admission). A smaller proportion of residents aged 65–74 years consulted an ophthalmologist (21%) or a cardiologist (18%) compared to the other age-groups. The proportion of residents 85 years and older consulting a neurologist (8% before admission, 5% after admission) was only half that observed in the other age-groups (16–17% before admission, 10% after admission). Only a small proportion of residents consulted a psychiatrist or neuropsychiatrist, but a higher proportion of younger residents consulted these specialists, and this proportion decreased significantly after nursing home admission (from 13% to 10% in 65–74 years age-group, from 8 to 6% in the 75–84 years age-group, stable at around 3% in residents 85 years and older). The proportion of residents who consulting a dentist was globally similar before and after nursing home admission, about 21%, but less than 9% of residents had consulted a dentist both before and after admission.

A particularly high proportion of residents received physical therapy after nursing home admission, increasing significantly from 43% to 64%, and for each age-group. The median number of sessions for residents receiving physical therapy increased from 32 per year before admission to 73 during the year after admission (mean: 47 before admission and 84 after admission).

### Hospitalizations

Although three-quarters of residents were hospitalized at least once during the year before nursing home admission, only 40% of residents were hospitalized during the year after admission (Table 3). The mean number of hospitalizations for all residents decreased significantly from 1.6 stays to 0.7 stays and those for residents who were hospitalized at least once decreased from 2.1 stays to 1.8 stays, the mean cumulative annual duration of all stays decreased from 21 days to 11 days and the proportion of emergency hospitalizations decreased from 60% to 52% of stays. In contrast, the frequency of hospitalizations without an overnight stay increased (12% to 22%). The proportion of emergency hospitalization increased with age, but hospitalizations without an overnight stay were less frequent in people 85 years and older. The mean number of hospitalizations for all residents decreased significantly for cerebral infarction, “symptoms, signs and abnormal clinical and laboratory findings, not elsewhere classified”, mental disorders, diseases of the musculoskeletal system. Besides, it increased significantly for heart failure, fracture of the femur, diseases of the respiratory system and of the digestive system. The main diagnosis with an increase of short-stay hospitalization proportion were injuries and poisoning, mental and behavioural disorders, factors influencing health status, disease of the eye (Table 4), bearing in mind that the number of hospitalizations decreased after nursing home admission. The majority of hospitalizations for these reasons were emergency admissions (70% and 79%, respectively).

**Table 3:** Short-stay hospitalizations the year before and the year after admission by sex and age

	Women			Men			Total
Age (years)	65–74	75–84	≥ 85	65–74	75–84	≥ 85	
*N*	526	3120	7601	481	1253	1906	14,887
Proportion of people with at least one hospitalization (%)							
Before and after	30.0	31.3	30.5	36.4	40.5	37.3	32.6
Before only	39.4	42.1	44.5	38.0	39.8	41.0	42.8
After only	7.0***	7.7***	7.7***	7.7***	6.5***	8.1***	7.6***
Mean number of stays per person							
Before	1,71	1,56	1,46	1,85	1,91	1,61	1,56
After	0,65***	0,71***	0,66***	0,95***	1,03***	0,81***	0,73***
Mean number of stays per person with at least one hospitalization							
Before	2.5	2.1	1.9	2.5	2.4	2.1	2.1
After	1.8**	1.8**	1.7*	2.1**	2.2**	1.8***	1.8***
Mean cumulative length of stay (days) of hospitalized residents							
Before	27.5	20.9	20.1	27.7	24.8	21.7	21.4
After	10.5***	10.2**	11.1***	11.6***	12.9***	10.8***	11.0***
Proportion of emergency hospitalizations (%)							
Before	52.3	56.1	63.5	54.8	56.8	60.5	60.1
After	41.7***	47.5***	58.0***	40.7***	42.3***	53.1***	52.0***
Proportion of hospitalizations without overnight stay (%)							
Before	18.4	14.6	10.2	12.6	13.5	9.2	11.8
After	25.7**	27.4***	17.9***	30.5***	26.2***	18.3***	21.7***

Comparison before and after admission ***p < 0.001, ** < 0.01,* *p* < 0.05

**Table 4:** Principal diagnoses of hospital stays the year before and the year admission and proportion of stays of less than 1 day and stays with emergency admission

Age (years)	Mean number of stays per person		<1 day		Emergency	
	Before	After	Before	After	Before	After
Number of stays	23,209	10,830				
	%	%	%	%	%	%
Diseases of the circulatory system	0,31	0,27	3.3	4.2	69.0	69.6
Heart failure	0,08	0,11***	2.5	1.4	77.8	80.5
Cerebral infarction	0,05	0,02***	1.7	2.1	81.6	82.1
Injury, poisoning and certain other consequences of external causes	0,30	0,27	3.9	9.5***	79.4	78.6
Intracranial injury	0,03	0,03	9.7	26.3***	87.2	86.5
Fracture of shoulder and upper arm	0,02	0,01	3.7	7.1	76.2	78.6
Fracture of the femur	0,09	0,12***	1.0	2.4*	81.4	82.1
Symptoms, signs and abnormal clinical and laboratory findings. Not elsewhere classified	0,29	0,16***	10.5	24.2***	71.7	61.5***
Ascites	0,00	0,01***	25.0	78.0**	62.5	9.8***
Other symptoms and signs involving the nervous and musculoskeletal systems	0,07	0,01***	4.0	23.1***	70.6	60.0
Other symptoms and signs involving cognitive functions and awareness	0,04	0,01***	16.6	44.8***	65.9	46.0***
Malaise and fatigue	0,04	0,02***	11.0	14.0	78.5	67.5*
Mental and behavioral disorders	0,24	0,09***	17.7	46.3***	50.3	21.6***
Alzheimer’s disease	0,07	0,03***	26.2	67.6***	39.6	7.4***
Alcohol-related disorders	0,01	0,00***	2.2	57.1***	81.3	28.6**
Depressive disorder	0,02	0,01***	13.6	32.3*	55.6	19.4***
Factors influencing health status and contact with health services	0,15	0,16**	45.6	66.7***	19.2	3.4***
Encounter for follow-up examination after completed treatment for conditions other than malignant neoplasm	0,03	0,05***	89.3	90.6	0.3	0.7
Encounter for care involving renal dialysis	0,00	0,01***	29.2	90.4***	NA	NA
Encounter for other aftercare and medical care	0,02	0,03***	57.0	47.1*	6.8	8.6
Diseases of the respiratory system	0,14	0,20***	2.3	3.8*	75.0	76.0
Pneumonia, unspecified organism	0,04	0,06***	2.6	4.0*	74.8	77.8
Other chronic obstructive pulmonary disease	0,02	0,03***	1.3	3.2	77.5	76.9
Respiratory failure. Not elsewhere classified	0,01	0,02***	1.6	3.8	78.6	75.2
Diseases of the musculoskeletal system and connective tissue	0,13	0,05***	3.7	9.2***	60.1	38.2***
Other disorders of muscle	0,04	0,00***	3.4	7.7*	84.0	84.6
Diseases of the digestive system	0,11	0,14***	10.7	10.8*	57.3	54.9
Paralytic ileus and intestinal obstruction without hernia	0,02	0,03***	3.9	4.1	77.6	78.7
Diseases of the nervous system	0,10	0,06***	13.3	24.5***	54.3	54.1
Parkinson’s disease	0,01	0,00	7.9	27.6**	45.2	27.6
Epilepsy	0,01	0,02	2.0	4.0	80.9	80.8
Diseases of the genitourinary system	0,07	0,07	4.9	6.6	58.8	60.4
Endocrine diseases, nutritional and metabolic	0,05	0,04	4.3	10.5***	63.4	59.8
Diseases of the eye and adnexa	0,05	0,11	58.8	80.0***	3.7	1.2**
Age-related cataract	0,04	0,09	65.3	83.3***	0.2	0.4
Neoplasms	0,05	0,07	16.7	32.3***	22.3	11.1***
Other malignant neoplasm of skin	0,01	0,02	51.2	60.1	3.7	1.4
Diseases of the blood and blood-forming organs and certain disorders involving the immune mechanism	0,03	0,06	9.4	9.0	52.0	45.5***
Certain infectious and parasitic diseases	0,03	0,03	1.7	5.2*	72.2	74.7
Diseases of the skin and subcutaneous tissue	0,02	0,03	8.8	21.3***	40.3	21.3***
Ulcer of lower limb, not elsewhere classified	0,01	0,01	10.6	5.7	42.6	22.9*

*NA* data not available for sample sizes <10

Comparison before and after admission ***p < 0.001, ** < 0.01,* *p* < 0.05

## Discussion

This study of reimbursed health care use, based on a cohort of about 15,000 residents admitted to EHPAD SNH during the first quarter of 2013 and still alive 1 year later, reveals a high prevalence of certain diseases, such as cardiovascular and neurovascular disease, which were present in one-half of all residents. Diseases prevalence and health care use changed considerably during the year following nursing home admission. For example, specialist consultations (at least once during the year) decreased (ophthalmologists: from 29 to 25%, cardiologists: from 27 to 21%), but physical therapy sessions increased in terms of both the proportion of residents (from 43 to 64%) and number of sessions (from an average 47 to 84 sessions). Dental consultations remained stable (21%). The proportion of residents hospitalized at least once during the year decreased, but remained high after nursing home admission (from 75 to 40%), and the mean cumulative duration of hospitalization also decreased (from 21 days to 11 days). In contrast, emergency hospitalizations decreased and the proportion of hospitalizations without an overnight stay doubled to reach 22% of all stays. The main reasons for admission to hospital were diseases of the circulatory system and injuries, while the proportion of mental and behavioral disorders and symptoms, signs and abnormal clinical and laboratory findings decreased after nursing home admission and the proportion of diseases of the respiratory system increased after nursing home admission.

The main diseases identified in EHPAD nursing home residents (cardiovascular diseases and dementia) are classically reported in institutionalized elderly people [5, 11]. The prevalence of stroke was higher in 2012 than in 2013, while stroke sequelae were more prevalent in 2013, which is consistent with the fact that stroke is a risk factor for institutionalization and was therefore identified more frequently in 2012 as a disease that may have justified nursing home admission [12]. The increased prevalence of these diseases following nursing home admission can be partly explained by an increased risk for onset or deterioration of these age-related diseases. The functional repercussions and dependence associated with these diseases are factors predisposing to institutionalization. This increased prevalence may also reflect more frequent screening or diagnosis at the critical time of nursing home admission or an increased prevalence related to management adaptations related to institutionalization, possibly allowing better identification of these diagnoses in the database (ALD applications, hospitalizations, treatments of certain diseases). However, disease prevalences are probably underestimated, as algorithms mainly based on ALD status or diagnoses coded during hospital stays for most diseases can only identify the most severely ill patients or those patients in situations in which ALD status would be beneficial (outpatient care, no private health insurance, etc.). Nevertheless, the 2011 EHPA survey on all SNH residents and not only new admissions, reported a similar prevalence for arrhythmias, conduction disorders (26%) and a slightly higher prevalence for coronary heart disease (18% vs 16%), stroke (18% vs 15%), and dementia (49% vs 47%). A low prevalence of depression (36% in the 2011 Ehpa survey vs 9% in our study) was observed in the present study, but 46% of people admitted to SNH used antidepressants [13]. The incidence or deterioration of health problems before SNH admission could generate SNH admission and may increase drug utilization [14]. For the nursing home residents of this study a 22% overall mortality in 2013 was found and, by comparison to people not residents in 2013 the standardized mortality ratio (SMR) was significanlty high for women (65–74 years: SMR = 15.9; 75–84 years: SMR = 5.8; 85 years and more: SMR = 2.4) and for men (65–74 years: SMR = 8.8; 75–84 years: SMR = 5.8; 85 years and more: SMR = 2.9) [15].

Almost one-half of all patients changed general practitioner during the year following nursing home admission, which can probably be partly explained by the distance of the nursing home from the patient’s own home. The French 2011 EHPA survey showed that only 30% of people remained in the same municipality when they entered the nursing home and that two-thirds were not admitted to the nursing home closest to their own home [16]. The change of general practitioner could also be related to the doctor’s failure to provide home visits or to the EHPAD organizational network. However, general practitioner consultations remained very frequent after nursing home admission, with an average of 15 consultations per year.

The frequency of specialist consultations decreased after nursing home admission. Specialist consultations are probably more complex as patients become less mobile. Nursing home health care coordination may also replace the need for specialist consultations, together with a change of management objectives in the context of a deteriorating disease or following the acute episode that justified nursing home admission. In the French Handicap-Santé survey, the frequency of specialist consultations decreased with the level of dependence and institutionalized patients consulted specialists less frequently than those living at home, with a comparable level of dependence [17]. Nevertheless, the low rate of neurologist and psychiatrist consultations, particularly among the oldest residents, despite the increased prevalence of neuropsychiatric disorders, reflects the difficulty of access to this type of specialist for the elderly.

In contrast, physical therapy use changed dramatically following nursing home admission with an increased proportion of residents concerned (from 43% to 64%) and especially a higher frequency of physical therapy sessions (from an average of 47 to 84 sessions during the year). The increased use of physical therapy is probably justified by the resident’s deteriorating state of health, and the diseases or their sequelae that justified nursing home admission. It could possibly be facilitated by the nursing home organization, as access to a physical therapist may be more difficult to organize at the elderly patient’s home. Physical therapy use was comparable to that observed in the French Handicap-Santé survey, which concerned one in every two dependent people, whether at home or in an institution [18].

While the Handicap-Santé survey showed that institutionalization was associated with decreased dental consultations (from 29% at home to between 18 and 25% in the institution), the present study showed that a stable proportion of residents consulted a dentist after nursing home admission (21%) [18]. However, in our study, the residents who consulted a dentist before nursing home admission differed from those who consulted a dentist after nursing home admission, as only 9% of residents consulted a dentist both before and after admission, which may reflect the detection of dental problems, as well as facilitated or more systematized access in the nursing home. Dental care is particularly important in this frail population due to the demonstrated links between oral health, nutrition, overall health and quality of life [19, 20].

Hospitalization rates were higher before nursing home admission. Hospitalization rates before nursing home admission include hospitalizations for reasons justifying nursing home admission. Longer hospital stays before nursing home admissions also probably reflect the severity of the disease justifying nursing home admission, but also the time required to organize post-discharge management. The proportion of residents hospitalized decreased markedly after nursing home admission, but nevertheless remained high: 40% of residents were hospitalized at least once during the year following admission with an increased rate of short-term management without an overnight stay and a reduction of emergency room management globally and for certain groups of diseases, such as mental and behavioral disorders. A review of the literature published in 2008 reported hospitalization rates of institutionalized people between 9 and 59% in North America, and a Swedish cohort study reported a hospitalization rate of 46% over a 3-year period, including 25% during the first 6 months, but these rates vary according to the definitions of hospitalization, the health system and the level of care provided by SNH, which also influences the types of diagnoses managed [21, 22]. A French study conducted in the Midi-Pyrénées region revealed that almost one-third of residents were hospitalized over a one-year period [23]. The majority (60%) of hospitalizations in our study were emergency admissions prior to nursing home admission; this rate decreased to 52% after nursing home admission, but the highest hospitalization rates were observed among the frailer residents 85 years and older. It is noteworthy that variable rates (fairly high, between one-third and one-half) of emergency room visits not followed by admission have been reported in various studies, but this aspect was not analyzed in the present study [24–26].

The main reasons for admission identified in this study, cardiovascular disease and injuries due to falls, are consistent with those reported in the literature, which also include respiratory and infectious diseases [22, 27, 28]. In France, the *Haute Autorité de Santé* (French National Authority for Health) has published guidelines designed to reduce nonelective hospitalizations of nursing home residents, especially by proposing prevention actions targeting falls and drug-induced iatrogenic disease, by promoting the nursing home treatment of certain diseases such as pneumonia, or by optimizing the mobilization of nursing home internal and external resources in order to limit unnecessary transfers to emergency rooms. Although EHPAD SNH are medicalized institutions, nursing staff is relatively limited and access to clinical pathology or radiology is also limited, sometimes making complete nursing home management relatively difficult. Management of certain situations in SNH would avoid the complications related to hospitalization, but also depends on nursing home organizational factors and coordination between health and medico-social systems. Nevertheless, the increased number of day-only stays, excluding treatment sessions, tends to suggest better utilization of geriatric services after nursing home admission, allowing implementation of more long-term prevention actions based on the assessments and recommendation of these day-only admissions.

This study was based on a large population composed of all national health insurance general scheme beneficiaries, i.e. about 69% of all people 65 years and older. People covered by other health insurance schemes according to their occupation probably present different socioeconomic, exposure and health care use characteristics. The target population of new nursing home admissions was limited to those residents who were still alive 1 year after admission in order to assess annual health care use, but to exclude terminal care, which probably comprises higher proportions of certain forms of care, such as hospitalization.

As diseases are not directly identified in the SNIIRAM database, multi-source algorithms have recently been developed and have been submitted to sensitivity analyses and expert review [10]. Although the method used in this study is not subject to the biases of self-reported surveys, it presents the classical limitations of data derived from medical and administrative databases. Nevertheless, these comprehensive data are derived from homogeneous data collection, but only concern individual reimbursements, requiring selection of subpopulations according to the nature of the study. This database only concerns diseases and health states managed by the health care system and therefore identified by the patient and by nursing staff. This study cannot reliably assess the correlation between health care use and disease status, as the precise impact of the disease, its degree of severity and the level of dependence are unknown. However, concordances were observed between the prevalences of diseases before and after nursing home admission, and health care professional consultations and reasons for admission. One of the limitations of the databases analyzed in this study is the absence of information concerning dependence and family support. However, this limitation could be overcome if, in the future, these databases were enriched by indicators of dependence, allowing evaluation of the autonomy of elderly people in activities of daily living.

## Conclusion

Linking of RESID-EHPAD and SNIIRAM databases now allows us to study and monitor trends in the characteristics of nursing home residents, their disease status and their health care use. The data currently available in databases cannot be used to evaluate the relevance of the various types of health care use. Nevertheless, this study demonstrates marked changes in health care use following nursing home admission. Nursing home admission is associated with a reduction of some forms of outpatient care and hospitalizations. Hospitalization remained frequent, but with shorter stays and less frequent emergency admissions, and a higher proportion of hospitalizations without overnight stays. Specialist consultations decreased, dental consultations remained globally stable and physical therapy use markedly increased.

## Additional file

Additional file 1: Algorithms for identifications of chronic diseases, health events, or chronic treatments from the French National Health Insurance system. (DOC 102 kb)

## Abbreviations

APA: *Allocation Personnalisée d’Autonomie* (APA) (dependent person’s pension)
CNAM-TS: Caisse Nationale d’Assurance Maladie fund
EHPAD: *Établissements d’Hébergement pour Personnes Âgées dépendantes [*nursing homes for dependent elderly people]
PMSI: *Programme de médicalisation des systèmes d’information* (PMSI) [Medical Information System Programme]
RESID-EHPAD: tool allowing nursing homes to automatically transmit the lists of their residents with admission and discharge dates to French national health insurance
SNIIRAM: *Système National d’Information Interrégimes de l’Assurance Maladie* [National Health Insurance Information System]
