# Supplementary material for: Impact of nursing home admission on health care use and disease status elderly dependent people one year before and one year after skilled nursing home admission based on 2012–2013 SNIIRAM data
Source: BMC Health Serv Res. 2017 Sep 18;17:667. doi: 10.1186/s12913-017-2620-6 (PMC5604505; doi:10.1186/s12913-017-2620-6)
Supplement: Additional file 1: — Algorithms for identifications of chronic diseases, health events, or chronic treatments from the French National Health Insurance system. (DOC 102 kb) [file 12913_2017_2620_MOESM1_ESM.doc]

**Additional file 1 :** Algorithms for identifications of chronic diseases, health events, or chronic treatments from the French National Health Insurance system

| **Conditions** | **Information sources and historical depth in algorithms for year t^1^** | | | |
| --- | --- | --- | --- | --- |
| **Defined algorithms** | **Hospital discharge diagnoses (ICD-10 codes)** | **LTD diagnoses^2^ (ICD-10 codes)** | **Pharmacy  reimbursement claims (ATC codes)** | **List of ICD-10 codes**  **included in algorithms^3^** |
| ***Cardiovascular and cerebrovascular disease*** |  |  |  |  |
| Acute ischemic heart disease | year t | - | - | I21-I24 |
| Chronic ischemic heart disease or history of acute ischemic heart disease^4^ | years t to t-4 | year t | - | I20-I25 |
| Acute cerebrovascular disease (excluding transient attacks) | year t | - | - | I60-I64 |
| Sequelae of cerebrovascular disease of history of acute cerebrovascular disease^4^ | years t to t-4 | year t | - | I60-I64, I67-I69, G81 |
| Heart failure with inpatient care within year | year t | - | - | I50, I11.0, I13.0, I13.2, I13.9, K76.1, J81 |
| Heart failure without inpatient care within year^4^ | years t to t-4 | year t | - | I50, I11, I13, I11.0, I13.0, I13.2, I13.9, K76.1, J81 |
| Peripheral vascular disease | years t to t-4 | year t | - | I70, I73, I74, I70.2, I73.9, I74.0, I74.3, I74.4, I74.5 |
| Cardiac arrhythmias | years t to t-4 | year t | - | I44, I45, I47-I49 |
| Cardiac valve diseases | years t to t-4 | year t | - | I05-I08, I34-I39 |
| Pulmonary embolism | year t | - | - | I26 |
| Other cardiovascular diseases | - | year t | - | other codes mostly among I- (n=42) |
| ***Pharmacological CVD prevention*** |  |  |  |  |
| Antihypertensive drug therapy within year^5^ | - | - | year t | - |
| Pharmacological lipid therapy within year^5^ | - | - | year t | - |
| ***Diabetes*** | year t to t-1 | year t | year t to t-1 | E10-E14, G59.0, G63.2, G73.0, G99.0, H28.0,  H36.0, I79.2, L97, M14.2, M14.6, N08.3 |
| ***Cancer^3^ (including solid and hematological malignant neoplasms)*** |  |  |  |  |
| Female breast cancer | year t to t-1 | year t to t-1 | - | C50, D05 |
| History of female breast cancer^3^ | years t to t-4 | prior to year t-1 | - | C50, D05 |
| Colorectal cancer | year t to t-1 | year t to t-1 | - | C18-C20, D01.0, D01.1, D01.2 |
| History of colorectal cancer^3^ | years t to t-4 | prior to year t-1 | - | C18-C20, D01.0, D01.1, D01.2 |
| Lung cancer | year t to t-1 | year t to t-1 | - | C33, C34, D02.1, D02.2 |
| History of lung cancer^3^ | years t to t-4 | prior to year t-1 | - | C33, C34, D02.1, D02.2 |
| Prostate cancer | year t to t-1 | year t to t-1 | year t to t-1 | C61, D07.5 |
| History of prostate cancer^3^ | years t to t-4 | prior to year t-1 | - | C61, D07.5 |
| Other malignant neoplasms (including hematological) | year t to t-1 | year t to t-1 | - | other codes among C- or D00-D09 |
| History of other malignant neoplasms (including hematological)^4^ | years t to t-4 | prior to year t-1 | - | other codes among C- or D00-D09 |
| ***Psychiatric disease*** |  |  |  |  |
| Schizophrenia and delusional diseases | years t to t-4 | year t | year t | F20-F25, F28-F29 |
| Depression and mood diseases | years t to t-4 | year t | year t | F30-F34, F38-F45, F48 |
| Mental deficiency | years t to t-1 | year t | - | F70-F73, F78, F79 |
| Substance abuse disorders (drug, alcohol, cannabis) | years t to t-1 | year t | - | F10-F19 |
| Disorders of psychological development | years t to t-1 | year t | - | F80-F84, F88-F95, F98 |
| Other psychiatric and behavioural diseases | years t to t-1 | year t | - | other codes among F- (n=21) |

| **Defined algorithms** | **Hospital discharge diagnoses (ICD-10 codes)** | **LTD diagnoses^2^ (ICD-10 codes)** | **Pharmacy  reimbursement claims (ATC codes)** | **List of ICD-10 codes**  **included in algorithms^3^** |
| --- | --- | --- | --- | --- |
| ***Psycholeptic or psychoanaleptic drug treatment*** |  |  |  |  |
| Antidepressant or mood regulator drug therapy within year^6^ | - | - | year t | - |
| Antipsychotic drug therapy within year^6^ | - | - | year t | - |
| Anxiolytic drug therapy within year^6^ | - | - | year t | - |
| Hypnotic or sedative drug therapy within year^6^ | - | - | year t | - |
| ***Dementia and Neurological disease*** |  |  |  |  |
| Dementia (including Alzheimer's disease) | years t to t-4 | year t | year t to t-1 | F00-F03, F05.1, G30 |
| Parkinson disease | years t to t-4 | year t | year t | F02.3, G20 |
| Multiple sclerosis | years t to t-4 | year t | - | G35 |
| Paraplegia and tetraplegia | years t to t-4 | year t | - | G82 |
| Myopathic and myasthenic syndromes | years t to t-4 | year t | - | G70-G73 |
| Epilepsy | years t to t-4 | year t | - | G40, G41 |
| Other neurological diseases | - | year t | - | other codes (extensive list) |
| ***Chronic respiratory diseases (including asthma)*** | years t to t-4 | year t | year t | J40-J47, J96, J98 |
| ***Inflammatory and systemic diseases*** |  |  |  |  |
| Inflammatory bowel diseases | years t to t-4 | year t | - | K50, K51, M07.4, M07.5 |
| Rheumatoid arthritis and related arthropathies | years t to t-4 | year t | - | M05, M06, M08, M09 |
| Ankylosing spondylitis and related arthropathies | years t to t-4 | year t | - | M07, M08.1, M45, M46 |
| Systemic and connective tissue diseases | years t to t-4 | year t | - | L93-L94, M30-M36 |
| ***Rare diseases*** |  |  |  |  |
| Metabolic disorders or amyloidosis | years t to t-4 | year t | - | extensive list mostly among E- codes |
| Cystic fibrosis | years t to t-4 | year t | - | E84 |
| Hemophilia and coagulation defects | years t to t-4 | year t | - | D66-D69 |
| ***HIV^8^*** | years t to t-4 | year t | year t | B20-B24, F02.4, Z20.6, Z21 |
| ***End-Stage Renal Disease*** |  |  |  |  |
| Dialysis^4,8^ | years t to t-1 | - | - | - |
| Kidney transplant within year^8^ | year t | - | - | - |
| Post-transplant immunosuppressive drug treatment^4,8^ | years t to t-4 | year t | year t | - |
| ***Liver and pancreas diseases (including chronic and acute failures)*** | years t to t-4 | year t | year t | B18, I85, K70-K76, K85, K86, Z94.4 |
| LTDs non included elsewhere | - | year t | - | extensive list |
| Analgesics and anti-inflammatory drug treatment^7^ | - | - | year t | - |

^1^A detailed presentation of each algorithm in French is available at: <http://www.ameli.fr/fileadmin/user_upload/documents/Cartographie_des_pathologies__methodologie_detaillee.pdf>

^2^For beneficiaries under LTD (long term chronic disease) status

^3^For each algorithm, a specific subset of codes among the reported list is included by data source (LTD, principal or associated inpatient diagnoses).

^4^Algorithm subject to hierarchical rule, as follows: acute > chronic or sequelae; cancer > history of cancer; transplant > post-transplant treatment > dialysis

^5^Exclusive from: ischemic heart disease, cerebrovascular disease, heart failure, peripheral vascular disease, diabetes, end-stage renal disease

^6^Exclusive from: psychiatric disease

^7^Exclusive from any other algorithm

^8^Algorithm including outpatient biological procedures, medical procedures or Diagnosis-Related-Group information
